# Multinucleated Giant Cells: Current Insights in Phenotype, Biological Activities, and Mechanism of Formation

**DOI:** 10.3389/fcell.2022.873226

**Published:** 2022-04-11

**Authors:** Kourosh Ahmadzadeh, Margot Vanoppen, Carlos D. Rose, Patrick Matthys, Carine Helena Wouters

**Affiliations:** ^1^ Laboratory of Immunobiology, Department Microbiology and Immunology, Rega Institute, KU Leuven – University of Leuven, Leuven, Belgium; ^2^ Division of Pediatric Rheumatology Nemours Children’s Hospital, Thomas Jefferson University, Philadelphia, PA, United States; ^3^ Division Pediatric Rheumatology, UZ Leuven, Leuven, Belgium; ^4^ European Reference Network for Rare Immunodeficiency, Autoinflammatory and Autoimmune Diseases (RITA) at University Hospital Leuven, Leuven, Belgium

**Keywords:** multinucleated giant cell (MGC), osteoclast, foreign body giant cell (FBGC), Langhans giant cell (LGC), macrophage, cell fusion, migration, multinucleation

## Abstract

Monocytes and macrophages are innate immune cells with diverse functions ranging from phagocytosis of microorganisms to forming a bridge with the adaptive immune system. A lesser-known attribute of macrophages is their ability to fuse with each other to form multinucleated giant cells. Based on their morphology and functional characteristics, there are in general three types of multinucleated giant cells including osteoclasts, foreign body giant cells and Langhans giant cells. Osteoclasts are bone resorbing cells and under physiological conditions they participate in bone remodeling. However, under pathological conditions such as rheumatoid arthritis and osteoporosis, osteoclasts are responsible for bone destruction and bone loss. Foreign body giant cells and Langhans giant cells appear only under pathological conditions. While foreign body giant cells are found in immune reactions against foreign material, including implants, Langhans giant cells are associated with granulomas in infectious and non-infectious diseases. The functionality and fusion mechanism of osteoclasts are being elucidated, however, our knowledge on the functions of foreign body giant cells and Langhans giant cells is limited. In this review, we describe and compare the phenotypic aspects, biological and functional activities of the three types of multinucleated giant cells. Furthermore, we provide an overview of the multinucleation process and highlight key molecules in the different phases of macrophage fusion.

## Introduction

Cell-cell fusion is the process in which the outer plasma membranes of two cells merge together forming a new syncytial cell (Pereira et al., 2018). In mammalians, both homotypic and heterotypic cell-cell fusion are crucial to multiple physiological processes (Skokos et al., 2011). It is essential for reproduction through fusion of a sperm cell with an oocyte, resulting in a diploid zygote (Georgadaki et al., 2016), development of the embryo (fusion of cytotrophoblast cells into the syncytiotrophoblast, required for proper functioning of the placenta) (Ma et al., 2020), development of muscle fibers (fusion of myoblasts into myofibrils) (Deng et al., 2017), and tissue repair (e.g. neuronal cell-cell fusion during axonal regeneration) (Giordano-Santini et al., 2016). However, cell-cell fusion may also contribute to pathological conditions including homotypic cancer cell fusion in which cancer cells fuse with each other or heterotypic cancer cell fusion where cancer cells fuse with other cell types like macrophages, endothelial cells or stem cells (Strick et al., 2007; Xue et al., 2015; Fernandes et al., 2019).

Among all immune cell types, macrophages possess great ability to undergo fusion with themselves and with other cell types (Pereira et al., 2018). Macrophages are innate immune cells that belong to the myeloid cell lineage (Gentek et al., 2014; Varol et al., 2015). For many years, it was thought that all macrophages originate from adult hematopoiesis in bone marrow, giving rise to mature monocytes that circulate in the bloodstream. During inflammation, monocytes extravasate the blood vessels and enter the affected tissue where they differentiate into macrophages (Varol et al., 2015; Wynn and Vannella, 2016). This theory was revised when recent findings showed that many tissue-resident macrophages have an embryonic origin (Epelman et al., 2014; Gentek et al., 2014), developed through embryonic hematopoiesis in the yolk sac (Epelman et al., 2014; Gentek et al., 2014; Wynn and Vannella, 2016). Due to their longevity and capacity for self-renewal, these macrophages can populate various tissues with minimal contribution of monocyte-derived macrophages (Gentek et al., 2014; Varol et al., 2015).

Macrophages are highly versatile and modulate the immunological response from initiation to termination of inflammation and subsequent tissue repair (Shapouri-Moghaddam et al., 2018). To this end, macrophages adapt to the micro-environment and acquire diverse phenotypes with modified functions. Two main subtypes of macrophages are distinguished: classically activated macrophages M1 and alternatively activated macrophages M2 (Martinez et al., 2008; Atri et al., 2018). During T helper type 1 (Th1) immune responses, macrophages polarize towards M1 macrophages, which are pro-inflammatory in nature, characterized by their release of pro-inflammatory mediators (Martinez et al., 2008; Timmermans et al., 2016; Atri et al., 2018; Le and Crouser, 2018; Yunna et al., 2020). Moreover, M1 macrophages are capable of antigen presentation and pathogen clearance (Shapouri-Moghaddam et al., 2018). During Th2 immune responses, macrophages shift towards the M2 phenotype, which are involved in termination of inflammation, tissue homeostasis, and repair (Martinez et al., 2008; Shapouri-Moghaddam et al., 2018; Yunna et al., 2020). The immunomodulatory capacities are accomplished through production of anti-inflammatory mediators and removal of inflammatory triggers, such as pathogens and apoptotic cells, by phagocytosis (Shapouri-Moghaddam et al., 2018; Yunna et al., 2020).

Under certain circumstances, macrophages can fuse resulting in formation of multinucleated giant cells (MGCs) (Pereira et al., 2018). In general, these polykaryons are subdivided into three main subtypes: osteoclasts, foreign body giant cells (FBGCs), and Langhans giant cells (LGCs) (Anderson, 2000). Osteoclasts are found under physiological conditions in bone, where they function as bone resorbing cells (Novack and Teitelbaum, 2008; Drissi and Sanjay, 2016). However, osteoclasts are also involved in the pathogenesis of rheumatoid arthritis (RA) and osteoporosis, indicating that proper osteoclast regulation is crucial to prevent pathological conditions (Drissi and Sanjay, 2016). By contrast to osteoclasts, FBGCs and LGCs are exclusively found under pathological conditions. FBGCs are formed during inflammatory reactions against foreign material, including implants and protheses (McNally and Anderson, 2002; Jay et al., 2007). LGCs are part of granulomas, which are focal clusters of immune cells (Zumla and James, 1996), in both infectious and non-infectious diseases (Wang et al., 2020).

Although MGCs have been described over 150 years ago (Mizuno et al., 2001; Pagán and Ramakrishnan, 2018), our understanding of these cells, except from osteoclasts, has not been further improved. Whereas osteoclasts are well-characterized, little knowledge is available on the phenotype and functionality of FBGCs and LGCs. In this respect, it is worth mentioning that in literature, FBGCs and LGCs are often seen or regarded as one cell type, making it difficult to obtain good understanding of their characteristics and biological activities. Furthermore, the macrophage fusion process remains poorly understood, especially for LGCs. In the first part of this review, we focus on the phenotypic characteristics of distinct MGC subtypes and on the current knowledge of MGC function and biological activity. In the second part, we provide an overview of the mechanisms of MGC formation with a focus on the key players in the different fusion steps. Finally, a general conclusion is drawn and remaining questions are discussed.

## Osteoclast Phenotype, Functions, and Biological Activities

Osteoclasts are well-known as bone resorbing cells contributing to skeletal remodeling and homeostasis throughout life (Novack and Teitelbaum, 2008; Drissi and Sanjay, 2016). During active bone resorption, osteoclasts are characterized by a ruffled border facing the bone surface and the nuclei are located close to the apical membrane, opposite to the ruffled border (Novack and Teitelbaum, 2008; Brooks et al., 2019). Today, increasing evidence is found that osteoclasts act in multiple processes beyond bone resorption. In the next sections, we will focus on the contribution of osteoclasts in bone resorption, promotion of bone formation, vasculogenesis, and immune regulation.

### Bone Resorption

**FIGURE 1:**
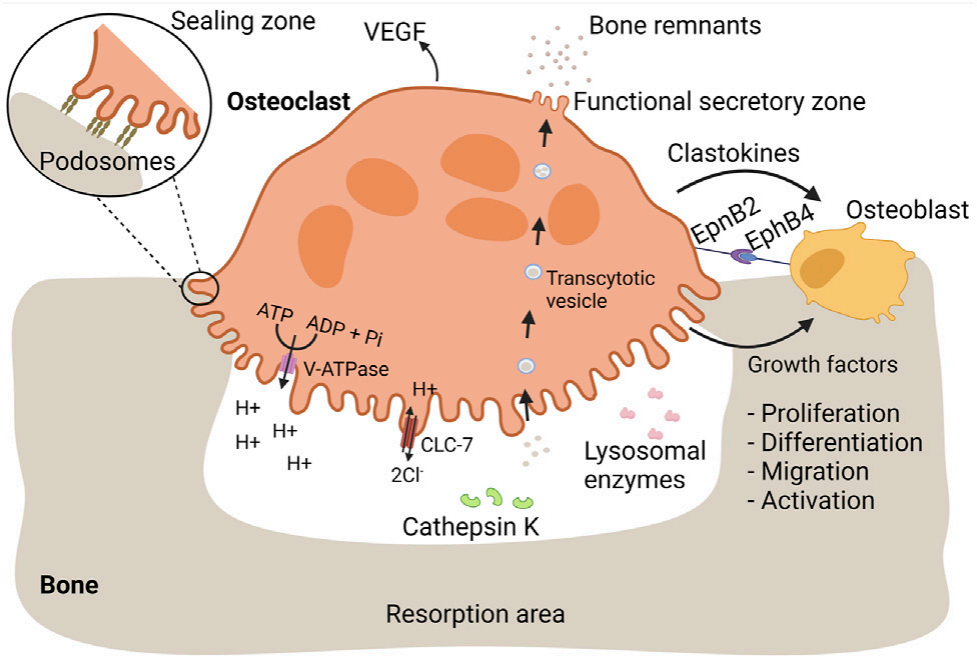
Osteoclasts mediate bone resorption and proper bone replacement by osteoblasts. Bone is a dynamic tissue that is remodeled by interplay of bone-resorbing osteoclasts and bone-generating osteoblasts. During bone resorption, osteoclasts firmly adhere to bone mediated by podosomes. Each podosome is composed of a central core of a dense F-actin network and actin polymerization activators. The core is surrounded by a loose F-actin network interspersed with regulatory proteins and adaptor proteins linking the podosome structure with integrins, referred as the podosome cloud. Podosomes are organized in an extensive circular pattern, the sealing zone, isolating an extracellular compartment in which bone resorbing substances are released. Bone-resorbing osteoclasts are characterized by a ruffled border, essential for bone resorption. The ruffled border is enriched with V-ATPase proton pumps, pumping hydrogen protons into the resorption lacunae, required for dissolution of bone minerals. During proton secretion, electroneutrality is maintained by release of chloride ions through CLC-7. Lysosomal enzymes, including cathepsin K, are released at the ruffled border and mediate the degradation of bone proteins, such as collagen I. Bone remnants are taken up in transcytotic vesicles and transported across the osteoclast cytoplasm towards the functional secretory zone where they are released. In order to ensure proper bone replacement, osteoclasts stimulate bone formation by osteoblasts. During bone resorption, osteoblastic growth factors are released from the bone matrix. Additionally, osteoclasts secrete clastokines, soluble factors that support osteoblast proliferation, differentiation, migration, activity, and survival. EpnB2 is expressed on osteoclast and is a transmembrane protein that stimulates osteoblast differentiation through interaction with EphB4 on osteoblasts. Vasculogenesis is stimulated through the release of VEGF.

Bone is a dynamic tissue consisting of an organic phase, which is predominantly type I collagen, and an inorganic phase, mainly composed of minerals in the form of hydroxyapatite (Buck and Dumanian, 2012). Throughout life, bone is remodeled by interplay of bone resorbing osteoclasts and osteoblasts generating new bone tissue (Chiu et al., 2012; Chen et al., 2018). Osteoclasts are equipped with a specialized bone-resorbing mechanism, resulting in external degradation of bone tissue and subsequent uptake of bone remnants for further processing (Mulari et al., 2003; Cappariello et al., 2014). An overview of the osteoclastic bone resorption process is depicted in Figure 1.

#### Extracellular Bone Degradation

During bone resorption, osteoclasts firmly adhere to bone tissue which is mediated by specialized actin-based membrane extensions, called podosomes (Touaitahuata et al., 2013). Each podosome is composed of a central core of a dense F-actin network and actin polymerization activators (Jurdic et al., 2006). The core is surrounded by a loose F-actin network interspersed with regulatory proteins and adaptor proteins linking the podosome structure with integrins, referred as the podosome cloud (Jurdic et al., 2006; Georgess et al., 2014). At the onset of bone resorption, podosomes are organized into an extensive circular pattern: the sealing zone (Georgess et al., 2014), which is of great importance and essential for bone resorption (Jurdic et al., 2006). It defines an isolated extracellular compartment, the resorption lacunae, where bone tissue is degraded (Helming and Gordon, 2009). Therefore, the resorption lacunae could be seen as an extracellular lysosome, able to destroy bone matrix (Vignery, 2005a; Helming and Gordon, 2009). Since bone is too large to internalize, the formation of this extracellular lysosome is a crucial step in bone resorption (Cappariello et al., 2014).

Active bone-resorbing osteoclasts are characterized by a ruffled border facing the bone surface (Novack and Teitelbaum, 2008; Brooks et al., 2019). By contrast, LGCs and FBGCs do not have a ruffled border and are not considered to resorb bone, illustrating the importance of the ruffled border in bone resorption. This border originates from fusion of lysosomes or storage of granule-like structures with the plasma membrane, enabling the insertion of lysosomal membrane proteins into the plasma membrane and the release of lysosomal enzymes into the resorption lacunae (Stenbeck, 2002; Na et al., 2020).

##### Resorption of the Inorganic Bone Fraction

Resorption of the mineral bone phase is mediated by vacuolar (V)-ATPase proton pump, a lysosomal protein inserted into the ruffled border during bone resorption (Stenbeck, 2002). V-ATPase mediates the release of hydrogen protons in the resorption lacunae, leading to the dissolution of bone minerals (Väänänen et al., 1990; Supanchart and Kornak, 2008). During proton secretion, electroneutrality is maintained by chloride channel 7 (ClC-7) as it allows the release of chloride ions into the extracellular environment (Sørensen et al., 2007). Pharmacological inhibition of V-ATPase diminishes the acidification of the resorption lacunae and bone resorption (Woo et al., 1996; Karsdal et al., 2005; Sørensen et al., 2007), illustrating the importance of V-ATPase in bone degradation. Additionally, patients with mutations in osteoclastic V-ATPase a3 subunit or in ClC-7, suffer from osteopetrosis, a condition characterized by increased bone density, due to impaired acidification and bone resorption (Karsdal et al., 2005, 2007).

##### Resorption of the Organic Bone Fraction

Once the mineral fraction is resolved, osteoclasts switch their degrading capacities towards the organic bone phase (Blair et al., 1986). In order to break down bone proteins, osteoclasts release several lysosomal enzymes, such as proteases, acid phosphatases and hydrolases (Na et al., 2020).

###### Cathepsin K

Cathepsin K is a cysteine protease able to cleave collagen type I, the most abundant protein in bone (Zaidi et al., 2001; Kiesel et al., 2009; Gradin et al., 2012; Drake et al., 2017). A major role for cathepsin K in breakdown of bone matrix proteins has been illustrated by multiple findings. In activated osteoclasts, cathepsin K is highly enriched at the ruffled border (Zaidi et al., 2001). In addition, absence of cathepsin K in humans leads to pycnodysostosis, a heritable disease characterized by osteopetrosis and a short stature (Gelb et al., 1996; Zaidi et al., 2001; Drake et al., 2017). Likewise, cathepsin K knock-out (KO) mice develop osteopetrosis (Saftig et al., 1998; Zaidi et al., 2001). Finally, pharmacological inhibition of cathepsin K reduces extensive bone resorption and can be used as a treatment of osteoporosis (Novinec and Lenarčič, 2013).

###### Matrix Metalloproteases

Matrix metalloproteases (MMPs) have been implicated in the resorption process as well (Stenbeck, 2002). This notion is supported by the localization of MMP1, MMP9 and MT1-MMP in the ruffled border. However, bone resorption by osteoclasts derived from MMP9 or MT1-MMP KO mice is not abrogated, suggesting that these MMPs are not crucial for osteoclast activity. It is worth noting that the involvement of MMPs in bone resorption depends on the anatomical location of osteoclasts (Everts et al., 1999, 2006). Everts et al. reported that MMP inhibitors affect bone-resorbing activity of cranial osteoclasts, whereas the bone-degrading capacities of long bone osteoclasts remain unaltered (Everts et al., 1999). By contrast, in conditions of suppressed cathepsin K expression, MMP inhibitors further deteriorate the bone resorption activity of long bone osteoclast, indicating that MMPs may partly compensate for cathepsin K deficiency (Everts et al., 2006).

###### Tartrate Resistant Acid Phosphatase

Tartrate Resistant Acid Phosphatase (TRAP) is a non-specific phosphatase (Fleckenstein and Drexler, 1997) that is extensively expressed in osteoclasts (Kirstein et al., 2006). TRAP secretion is positively correlated with bone resorption (Kirstein et al., 2006; Hayman, 2008) and serum level of TRAP is often used as a biomarker for bone resorption (Kirstein et al., 2006; Mira-Pascual et al., 2020). Mice lacking TRAP display mild osteopetrosis and *in vitro* bone resorption is impaired in osteoclasts derived from these mice (Hayman et al., 1996; Hayman, 2008). Additionally, transgenic mice overexpressing TRAP show mild osteoporosis, further pointing towards a role for TRAP in bone resorption (Angel et al., 2000). TRAP has been detected in transcytotic vesicles (Mira-Pascual et al., 2020), referring to vesicles that originate from endocytosis of bone degradation products by osteoclasts (Madel et al., 2019). Within these vesicles, TRAP contributes to the degradation bone remnants by catalyzing the production of reactive oxygen species (ROS) that in turn damages the internalized bone proteins (Halleen et al., 1999; Halleen et al., 2003).

#### Intracellular Degradation

As mentioned before, osteoclasts are able to internalize bone remnants for intracellular degradation (Stenbeck, 2002; Mulari et al., 2003; Madel et al., 2019). Lysosomal enzymes are secreted at the periphery of the ruffled border, whereas uptake of degradation products takes place at the center (Mulari et al., 2003). The internalization is coordinated by the regular clathrin-mediated endocytosis machinery (Mulari et al., 2003) and results in the formation of intracellular vesicles filled with bone remnants: transcytotic vesicles (Madel et al., 2019). By transcytotic trafficking, vesicles are transported to the functional secretory zone (i.e., the membrane in opposite of the ruffled border) where the content is released into the extracellular environment.

### Promotion of Bone Formation

A tight regulation between osteoclast and osteoblast activity is crucial to maintain healthy bone tissue over time (Chen et al., 2018). Hyperactivation of osteoclasts leads to a condition characterized by low bone density and an increased risk of fractures, called osteoporosis (Armas and Recker, 2012), whereas osteoclast insufficiency may induce osteopetrosis (Stark and Savarirayan, 2009). In order to keep the right balance between bone resorption and formation, osteoclasts and osteoblasts mediate each other’s activity (Chen et al., 2018). Whereas osteoblasts modulate osteoclast differentiation and function, several osteoclastic mechanisms regulate osteoblast differentiation (Matsuo and Irie, 2008; Teti, 2013; Chen et al., 2018).

The bone matrix forms a reservoir for several growth factors, including transforming growth factor (TGF)-β, insulin like growth factor (IGF)-I, and bone morphogenic proteins (BMPs) (Matsuo and Irie, 2008; Teti, 2013). During bone resorption, these factors are released from the bone matrix and become activated through the acidic pH and/or enzymatic cleavage within the resorption lacunae (Teti, 2013). The activated growth factors in turn enhance osteoblastic bone formation, guaranteeing proper replacement of old bone tissue (Matsuo and Irie, 2008).

#### Clastokines

Osteoclasts also stimulate bone formation directly through the release of various soluble osteogenic factors, called clastokines (Lotinun et al., 2013). Amongst others, sphingosine-1 phosphate (SP-1 P), BMP6, TRAP, platelet-derived growth factor (PDGF), and hepatocyte growth factor (HGF) are well-described clastokines (DiGiovanni et al., 2012; Teti, 2013; Cappariello et al., 2014). SP-1 P is a signaling sphingolipid that enhances bone regeneration through multiple mechanisms (Higashi et al., 2016; Meshcheryakova et al., 2017). SP-1 P recruits osteoblast precursors (Meshcheryakova et al., 2017), promotes osteoblast proliferation, differentiation, activity, and survival (Higashi et al., 2016; Meshcheryakova et al., 2017). BMP6 induces osteoblast differentiation (Demirtaş et al., 2016) and bone mineralization (Luo et al., 2011; Zhu et al., 2012). In addition to its role in bone resorption, TRAP acts as a clastokine by facilitating osteoblast proliferation and differentiation (Gradin et al., 2012). PDGF acts as a chemotactic and mitogenic factor for osteoblasts (DiGiovanni et al., 2012), whereas HGF facilitates osteoblast proliferation and differentiation (Hossain et al., 2005; Frisch et al., 2016). Together, these factors facilitate the migration of osteoblast precursors, osteoblast proliferation, differentiation, and/or activity (Gradin et al., 2012; Cappariello et al., 2014).

Osteoclasts also promote bone formation through direct cell contact with osteoblasts, mediated by members of the Eph receptor family and their ephrin ligands (Zhao et al., 2006; Edwards and Mundy, 2008; Matsuo and Otaki, 2012; Tamma and Zallone, 2012). Eph receptors are tyrosine kinase receptors that are involved in multiple biological processes, including neuronal development (Kania and Klein, 2016). Interaction between Eph receptors and ephrin ligands results into bi-directional signaling, meaning that signaling pathways are activated in both receptor-expressing and ligand-expressing cells (Edwards and Mundy, 2008; Kania and Klein, 2016). Signal transduction from ephrin ligands to Eph receptors is referred as forward signaling, whereas reverse signaling points to signal transduction from Eph receptors to ephrin ligands (Kania and Klein, 2016). Several reports indicate that ephrinB2 ligand and EphB4 receptor are involved in osteoclast-osteoblast intercommunication (Zhao et al., 2006; Edwards and Mundy, 2008; Matsuo and Otaki, 2012; Tamma and Zallone, 2012). Differentiating and mature osteoclasts express ephrinB2, whereas osteoblasts express EphB4 (Zhao et al., 2006; Edwards and Mundy, 2008; Matsuo and Otaki, 2012). Forward signaling stimulates osteoblast differentiation (Zhao et al., 2006; Edwards and Mundy, 2008; Matsuo and Otaki, 2012), facilitating the formation of new bone tissue, whereas reverse signaling inhibits osteoclast maturation (Zhao et al., 2006). The combined action of both signaling cascades ensures efficient bone replacement by mediating simultaneously cessation of bone resorption and generation of new bone tissue (Edwards and Mundy, 2008).

### Vasculogenesis

Mounting evidence is emerging that osteoclasts and their precursors stimulate angiogenesis (Kiesel et al., 2007; Drissi and Sanjay, 2016; Han et al., 2018). It has been observed that bone vascularization and ossification is disturbed in the absence of osteoclasts (Matsuo and Otaki, 2012). Vascularization is important during bone development for the migration of bone cells involved in degradation of cartilage and subsequent bone deposition (Tombran-Tink and Barnstable, 2004). Furthermore, bone remodeling and repair depend on well-developed vascular networks (Grosso et al., 2017) to provide bone tissue with oxygen and nutrients (Kiesel et al., 2007; Grosso et al., 2017), to remove bone remnants (e.g. calcium and phosphate ions) (Kiesel et al., 2007; Grosso et al., 2017), and to recruit hematopoietic stem cells, bone cells and immune cells (Grosso et al., 2017). Various pro-angiogenic factors such as endothelial growth factor and PDGF isoform BB are upregulated during osteoclastogenesis (Kiesel et al., 2007), with some remaining expressed in mature osteoclasts (Tombran-Tink and Barnstable, 2004). Vascular endothelial growth factor (VEGF) (Tombran-Tink and Barnstable, 2004; Kiesel et al., 2007; Grosso et al., 2017), a key regulator of angiogenesis (Grosso et al., 2017), induces migration, proliferation, and survival of endothelial cells (Grosso et al., 2017; Han et al., 2018), whereas PDGF isoform BB (PDGF-BB) (Xie et al., 2014; Gao et al., 2017) promotes the recruitment of endothelial progenitor cells (Xie et al., 2014; Gao et al., 2017; Han et al., 2018) and their differentiation into mature endothelial cells (Gao et al., 2017). The pro-angiogenic action of PDGF-BB probably involves the induction of VEGF in endothelial progenitor cells (Gao et al., 2017), which in turn stimulates vessel formation. Furthermore, ephrinB2 regulates VEGF-induced endothelial cell migration and angiogenesis (Sawamiphak et al., 2010; Wang et al., 2010). Since osteoclasts express ephrinB2, it is tempting to hypothesize that osteoclasts also mediate angiogenesis through ephrin2B (Matsuo and Otaki, 2012). Next to direct stimulation of vessel formation, osteoclasts establish the required space for blood vessels to grow (through bone-resorption) (Grüneboom et al., 2019). To provide bone marrow of sufficient oxygen, nutrients and to establish efficient emigration of newly generated blood cells from the bone marrow to the external circulation, a well-established vascular network is indispensable. Therefore, a multitude of trans-cortical vessels cross the bone shaft in order to connect the bone marrow with the external circulation. The transcortical vessels require small channels through or small channels in the bone tissue, which are maintained by osteoclasts.

### Immunomodulation

It has been established that bone and the immune system are interconnected (Okamoto and Takayanagi, 2019). Hematopoietic stem cells and immune cell precursors originate from bone marrow (Okamoto and Takayanagi, 2019; Guder et al., 2020). Several signaling molecules, including cytokines, receptors, and transcription factors, are shared between the skeletal and immune system (Okamoto and Takayanagi, 2019). Also, many auto-immune and inflammatory disorders are characterized by bone destruction, including RA and Crohn’s disease (Veauthier and Hornecker, 2018; Madel et al., 2019; Ashai and Harvey, 2020). At last, a spectrum of bone alterations has been described in several genetically defined autoimmune conditions, caused by mutations in innate immune components (Bader-Meunier et al., 2018).

**FIGURE 2:**
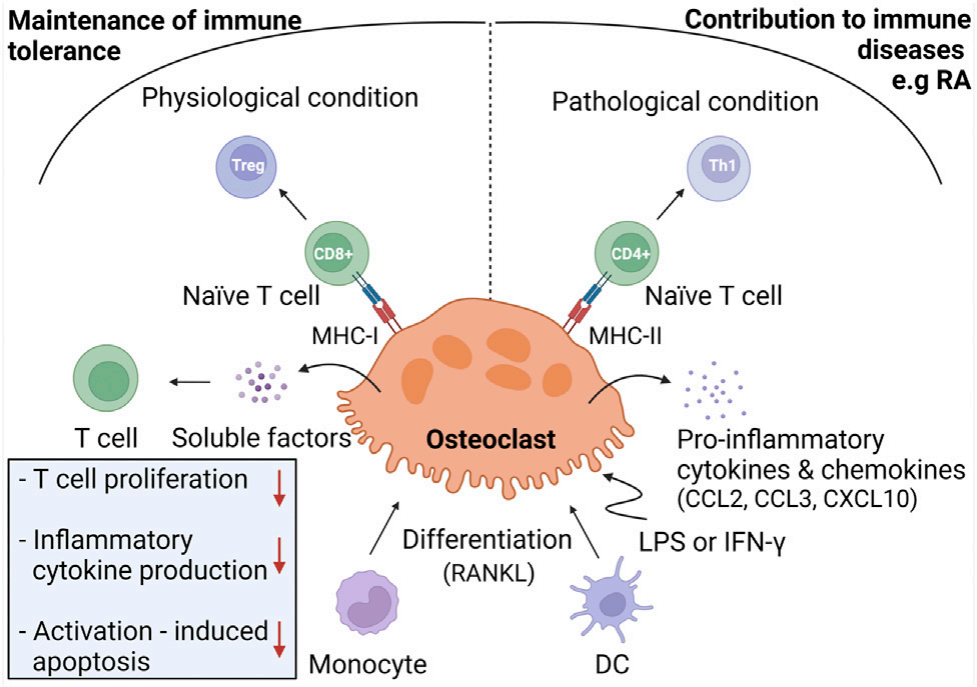
Immunoregulation by osteoclasts. Under physiological conditions, osteoclasts are considered to be derived from monocyte progenitor cells and exhibit immunomodulatory activities in order to maintain immune tolerance to bone remnants. To do so, osteoclasts induce Treg cells through antigen presentation in MHC-I complexes towards CD8^+^ T cells. Osteoclasts inhibit T cell proliferation, production of inflammatory cytokines, and activation-induced apoptosis through release of soluble mediators. Under inflammatory conditions, osteoclasts are hypothesized to derive from DC and to contribute to immune diseases, including RA. In response to LPS or IFN-γ, osteoclasts produce pro-inflammatory mediators and differentiate naïve T cells into Th1 cells through antigen presentation in MHC-II molecules.

It has been demonstrated that immune cells mediate osteoclast differentiation and activity. For example, T cells are able to promote osteoclastogenesis through the expression of RANKL (Kiesel et al., 2009; Grassi et al., 2011; Shashkova et al., 2016). Osteoclasts can be differentiated from monocytes, macrophages, or dendritic cells (DCs) (Speziani et al., 2007; Nishida et al., 2018; Lou et al., 2019; Wu et al., 2019; Narisawa et al., 2021). Since these osteoclast precursors are well-known for their immunological activities, it would not be surprising that osteoclasts may participate in immunological processes as well. Recently, accumulating evidence has shown that osteoclasts indeed perform immunological tasks, including phagocytosis, antigen presentation, and T cell activation (Li et al., 2010; Meng et al., 2010). The role of osteoclasts within the immune system is depicted in Figure 2.

#### Phagocytosis

Phagocytosis is the internalization of particles larger than 0.5 µm (Hirayama et al., 2001) in size by plasma membrane-derived vesicles (Rosales and Uribe-Querol, 2017). Professional phagocytes, including macrophages and DCs, internalize foreign particles for degradation purposes or in advance of antigen preparation and presentation (Madel et al., 2019). Osteoclasts engulf bone remnants for the purpose of bone degradation through clathrin-mediated endocytosis (Mulari et al., 2003). Other than bone remnants, osteoclasts have been reported to internalize many other particles through phagocytosis, including latex, polymethylmethacrylate, and titanium (Wang et al., 1997; Meng et al., 2010; Madel et al., 2019). Whether phagocytosis of particles other than bone remnants influences bone resorption remains unclear as conflicting results have been reported (Wang et al., 1997; Meng et al., 2010). Wang et al. showed that osteoclasts still exert bone resorption after they have internalized latex or polymethylmethacrylate particles, whereas Meng et al. suggested that bone resorption decreases upon phagocytosis of titanium particles (Meng et al., 2010). Moreover, several reports indicate that osteoclasts are able to phagocytose apoptotic cells (Taniwaki and Katchburian, 1998; Bronckers et al., 2000; Boabaid et al., 2001; Cerri et al., 2003; Harre et al., 2012). Apoptosis is a sterile form of programmed cell death, such that it does not elicit an inflammatory response (Xu et al., 2019). During apoptosis, various molecules are exposed to the cell surface that label the cell as being apoptotic (Savill, 1997). These molecules are recognized by phagocytic receptors on phagocytes followed by the internalization of the apoptotic cell (Savill, 1997). Macrophages and DCs are well-equipped to clear apoptotic cells through phagocytosis (Harre et al., 2012). Moreover, Harre et al. reported that osteoclasts express multiple proteins that are involved in the engulfment of apoptotic cells, to a similar or even higher extend than macrophages and DCs (Harre et al., 2012). This is reflected by the high phagocytic capacity of osteoclasts for murine apoptotic thymocytes *in vitro*. In contrast to macrophages, osteoclasts internalized the cells independently from serum, indicating that they do not use opsonization or that they make the opsins themselves. *In vivo* evidence has also been provided that osteoclasts perform phagocytosis under physiological conditions (Taniwaki and Katchburian, 1998; Bronckers et al., 2000; Boabaid et al., 2001; Cerri et al., 2003). Osteoclasts are able to engulf apoptotic bone cells, including chondrocytes, osteocytes, and osteoblasts (Taniwaki and Katchburian, 1998; Bronckers et al., 2000; Boabaid et al., 2001; Cerri et al., 2003). Osteocytes and chondrocytes are tightly surrounded by bone and cartilage matrices respectively, making them difficult to reach by classical phagocytes, such as macrophages (Harre et al., 2012). Therefore, osteoclasts are crucial to remove dying bone cells to prevent chronic inflammation or autoimmunity. Not only the accessibility renders osteoclasts more suitable for clearance of dying bone cells, also the fact that they do not rely on serum-derived opsins is important since bone tissue is poorly vascularized.

#### Antigen Presentation and T Cell Activation

Growing evidence indicates that osteoclasts interact with T cells. In bone marrow, T cells are in close proximity to osteoclasts (Grassi et al., 2011), suggesting that osteoclasts attract T cells. This hypothesis is supported by the finding that osteoclasts secrete T cell-attracting chemokines, including CC chemokine ligand (CCL)2, CCL3, and CXCL10 (Kiesel et al., 2009; Grassi et al., 2011). Adhesion assays showed that osteoclasts are able to recruit and retain T cells (Grassi et al., 2011). Next to mediating chemotaxis, osteoclasts can also regulate T cell activity and differentiation. In order to become activated, naïve T cells need to be primed with antigens presented on the surface of antigen presenting cells (APCs) (Sprent, 2005). Several reports demonstrated that osteoclasts can act as APCs. Indeed, osteoclasts cross present exogenous antigens in MHC-I molecules to CD8^+^ T cells (Buchwald et al., 2013; Le Goff et al., 2013). Characterization of these CD8^+^ T cells revealed that osteoclasts induce a regulatory T cell (Treg) phenotype, thereby inducing an immune modulatory effect (Buchwald et al., 2013; Le Goff et al., 2013; Shashkova et al., 2016). In addition, osteoclasts have been reported to present antigens in MHC-II molecules to CD4^+^ T cells, which in turn differentiate into Th1 cells (Le Goff et al., 2013; Ibáñez et al., 2016) (Figure 2).

It should be noted that not all studies that have examined the APC function of osteoclasts reached the same conclusion. Kiesel et al. reported that osteoclasts cannot present antigens to CD4^+^ T cells since they do not express MHC-II molecules (Kiesel et al., 2009). By contrast, other reports demonstrated that osteoclasts do express MHC-II molecules (Li et al., 2010, 2014; Le Goff et al., 2013; Ibáñez et al., 2016) and that they are able to present antigens to CD4^+^ T cells (Li et al., 2010, 2014; Le Goff et al., 2013). Furthermore, conflicting results about the expression of co-stimulatory molecules have been reported (Kiesel et al., 2009; Li et al., 2010, 2014; Ibáñez et al., 2016). Kiesel et al. reported that osteoclasts only express CD80 but not CD86 (Kiesel et al., 2009), whereas other groups found that osteoclasts express both molecules (Li et al., 2010, 2014; Ibáñez et al., 2016). These discrepancies could be explained by the immune environment in which osteoclast-T cell interactions take place (Le Goff et al., 2013; Ibáñez et al., 2016). Under physiological conditions, osteoclasts exhibit an immune-modulatory phenotype, characterized by the secretion of anti-inflammatory cytokines, such as IL-10 (Ibáñez et al., 2016). These osteoclasts induce Tregs by antigen cross presentation to CD8^+^ T cells (Buchwald et al., 2013; Le Goff et al., 2013; Shashkova et al., 2016). Additionally, they secrete soluble factors that inhibit T cell proliferation, inflammatory cytokine production, and activation-induced apoptosis of T cells (Grassi et al., 2011). It is assumed that these immunomodulatory functions of osteoclasts are crucial to maintain immune tolerance to bone remnants in order to prevent autoinflammation (Ibáñez et al., 2016). Conversely, under inflammatory conditions, osteoclasts may exacerbate the ongoing inflammation. Upon stimulation with LPS or IFN-γ, MHC-II expression is upregulated in osteoclasts (Grassi et al., 2011; Le Goff et al., 2013), which leads to increased Th1 cell induction. Furthermore, osteoclasts merely produce pro-inflammatory cytokines (TNF-α, IL-1β, IL-6 and IL-23) and chemokines (CCL2, CCL5 and CCL7) in an inflammatory environment (Ibáñez et al., 2016).

The differences in osteoclast activity might be also explained by the different origin of osteoclasts in various milieus (Ibáñez et al., 2016). Under physiological conditions, osteoclasts are considered to be derived from monocytes, whereas under inflammatory conditions they may originate from immature DCs. Therefore, the inflammatory osteoclast can be seen as an intermediate between the monocyte-derived osteoclast and the DC and probably plays a role in many immune diseases, including RA (Narisawa et al., 2021).

Osteoclasts may also indirectly regulate the activity of immune cells (van Niekerk et al., 2018). Bone tissue forms a reservoir for calcium, phosphate and magnesium ions, which are released during bone resorption. These ions are crucial for many immune related processes, such as immune cell proliferation, chemotaxis, and signaling.

Taken together, osteoclasts exert multiple functions within the human body of which bone resorption is the best recognized (Drissi and Sanjay, 2016). Throughout the last decades, increasing evidence was found that osteoclasts cannot longer be regarded as solely bone-resorbing cells and that they contribute to multiple processes, including osteoblast stimulation, vasculogenesis and immune regulation (Le Goff et al., 2013; Cappariello et al., 2014; Drissi and Sanjay, 2016). Compared to osteoclasts, the functionality of FBGCs and LGCs is less well established, and will be discussed in the next two sections.

## Foreign Body Giant Cell Phenotype, Functions, and Biological Activities

**FIGURE 3:**
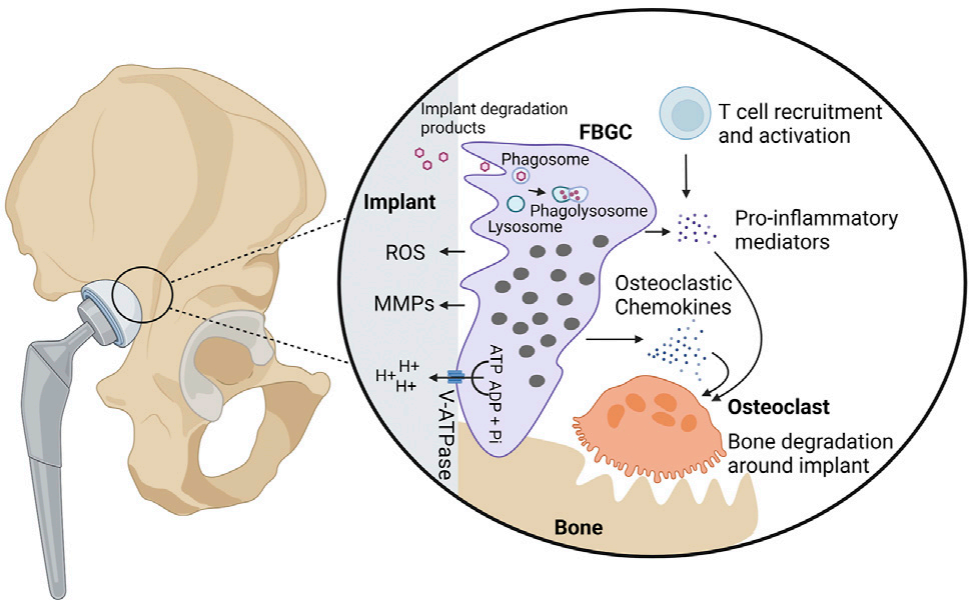
Biological activities of foreign body giant cells. FBGCs are formed on the surface of foreign bodies, including implants and protheses, and are thought to contribute to the degradation of foreign particles through phagocytosis or secretion of ROS, MMPs, and hydrogen protons. In case of bone implants, FBGCs are hypothesized to contribute to aseptic loosening, bone destruction around the implant. Hydrogen protons released from FBGCs dissolve bone minerals in proximity to the implant. Additionally, FBGCs recruit and activate osteoclasts through secretion of chemokines and pro-inflammatory cytokines. Finally, FBGCs are thought to recruit and activate T cells, which in turn produce osteoclast-stimulating mediators.

Among all MGC subtypes, FBGCs are the largest reaching up to 1 mm in size (Wang et al., 2020). They have an irregular shape and contain hundreds of nuclei, scattered throughout the cytoplasm (Anderson, 2000; Quinn and Schepetkin, 2009; Lemaire et al., 2012; Sakai et al., 2012; Wang et al., 2020). FBGCs are formed in foreign body reactions (FBRs), which are chronic inflammatory reactions against non-infectious foreign body material, including implants, protheses, and medical devices (McNally and Anderson, 2002; Anderson et al., 2008). FBRs involve a series of events that eventually leads to the formation of FBGCs and fibrotic encapsulation of the foreign body (Anderson et al., 2008; Sheikh et al., 2015). Upon implantation of biomaterial, host tissue is injured resulting into blood-material interactions. These interactions involve the adsorption of host proteins onto the surface of the biomaterial and thrombus formation at the tissue-material interface (Anderson et al., 2008). The deposited cloth of proteins is referred as the provisional matrix, which plays an important role in shaping the subsequent immunological reactions (Sheikh et al., 2015). The provisional matrix forms a docking site for immune cells and contains several bioactive agents, such as mitogens, cytokines, and chemokines, supporting chemotaxis, proliferation, and activation of immune cells. Among the recruited cell types, macrophages are prominent in shaping the FBR through production of immunological mediators (Anderson et al., 2008) and through their fusogenic capacity to form FBGCs over time in the presence of a conditioned cytokine milieu (Figure 3).

### Particle Degradation

It is assumed that FBGCs arise when individual macrophages are unable to remove foreign particles (Jay et al., 2010). In such conditions, macrophages fuse together in expectation that the bigger cell size and excess plasma membrane (Whitlock and Chernomordik, 2021) enable them to remove the threat (Han et al., 2000; Vignery, 2000; Milde et al., 2015).

#### Intracellular Degradation

One possible mechanism by which FBGCs may destroy their substrate is phagocytosis and intracellular degradation (Lee et al., 1981; Milde et al., 2015). Several researchers demonstrated that FBGCs are able to engulf particles that single macrophages cannot (Moreno et al., 2007; Milde et al., 2015). Milde et al. showed that FBGCs are more efficient in phagocytosing beads of 20 µm compared to macrophages and that beads of 45 µm are exclusively taken up by FBGCs (Milde et al., 2015). In addition, Moreno et al. showed that fusion of macrophages into FBGCs is crucial to be able to phagocytose 25 µm beads while IL-4 stimulated macrophages failed to phagocytose these beads. (Moreno et al., 2007). Inhalation experiments in rats, hamsters and guinea pigs provided *in vivo* evidence that alveolar macrophages preferably internalize small dust fibers (i.e. particles smaller than 5 µm in size), whereas particles larger than 10 µm are phagocytosed by FBGCs (Lee et al., 1981).

Protein adsorption on biomaterial surfaces plays probably an important role in the phagocytic properties of FBGCs. Milde et al. found that FBGCs are highly phagocytic for complement-opsonized particles (Milde et al., 2015). Indeed, the excessive phagocytic capacity was diminished when medium was depleted of complement component 3 (C3) or when FBGCs did not express the complement receptor 3 subunit CD11b. Complement-mediated phagocytosis is very efficient in FBGCs since the complement receptor 3 (CR3) localizes into membrane rumples, which provide excess cellular membrane to engulf large particles. Complement opsonization may be of great importance in phagocytosis of foreign bodies, such as polymer or metal particles, that do not have any ligands for phagocytic receptors. Since the nature of the biomaterial highly influences protein adsorption (Anderson et al., 2008), it can be speculated that it also interferes with the phagocytic capacity of FBGCs. Further research is required to investigate whether other phagocytic receptors are involved in the internalization of foreign bodies and whether the contribution of the receptors is altered by the nature of the biomaterial.

#### Extracellular Degradation

Next to phagocytosis, FBGCs destroy foreign bodies through the secretion of ROS and matrix metalloproteases (MMPs) (Jones et al., 2007). Release of these deleterious substances was found to lead to the failure of medical implants (Anderson et al., 2008), stressing the need for the development of resistant biomaterials (Ebert et al., 2005). Microscopic analyses revealed that FBGCs firmly adhere to their substrate thereby creating an isolated extracellular environment between the FBGC plasma membrane and the particle surface, called the sealing zone (ten Harkel et al., 2015; ten Harkel et al., 2016). The sealing zone can be regarded as an extracellular lysosome in which harmful substances are released in order to degrade the biomaterial (Vignery, 2005a). Evidence has been provided that FBGCs degrade collagen, which is often used as biomaterial for medical devices (van Wachem et al., 1991; Ye et al., 2011; ten Harkel et al., 2016). At sites where FBGCs are in close proximity to collagen bundles, the collagen structure loosens and fibrils look thinner, pointing to extracellular degradation (ten Harkel et al., 2016). FBGCs are also able to break down gelatin (i.e. denatured collagen) (Ye et al., 2011). The degradation of collagen and gelatin both rely on MMP13, an active collagenase and gelatinase. Furthermore, collagen and gelatin can be phagocytosed, suggesting that they undergo additional intracellular degradation.

Whether FBGCs preferably degrade their substrate internally (i.e. through phagocytosis) or externally (i.e. through secretion of ROS and MMPs) depends on the size of the particle (Sheikh et al., 2015). Existing data suggest that FBGCs efficiently phagocytose particles up to 100 µm in diameter. Though the phagocytic efficiency decreases with increased particle size and if needed, FBGCs could switch to external degradation.

### Aseptic Loosening

Peri-implant bone loss, referred as aseptic loosening, is often observed after implantation of biomaterials (Wang et al., 1997; Meng et al., 2010) and is considered as a main cause of implant failure (Wooley and Schwarz, 2004). After implantation, microparticles are often released from the bone implant and elicit a chronic inflammatory response (Gu et al., 2012; Goodman and Gallo, 2019). In case of a bone-anchored prothesis, the inflammatory reaction stimulates osteoclastogenesis and activity, thereby facilitating bone destruction around the implant (Wooley and Schwarz, 2004; Gu et al., 2012; Goodman and Gallo, 2019). In addition to osteoclasts, FBGCs are hypothesized to contribute to aseptic loosening by massive excess of hydrogen protons (ten Harkel et al., 2015). FBGCs, like osteoclasts, highly express the vacuolar V-ATPase proton pump which enables them to secrete massive amounts of hydrogen protons. Whether the release of protons by FBGCs contributes to bone destruction around implants *in vivo* needs to be elucidated. Apart from the possibility to dissolve bone minerals, FBGCs are suspected to promote aseptic loosening by stimulation of bone degradation by osteoclasts. FBGCs produce various osteoclastic chemokines, including CCL2, CCL3, and CCL9, which together promote osteoclast formation and survival (Khan et al., 2014).

### Shaping of the Foreign Body Reaction

FBGCs may contribute to the establishment and maintenance of foreign body reactions through the production of many inflammatory mediators. It has been reported that FBGCs express CCL2, CCL3, and CCL5, which are very potent macrophage chemoattractants (Khan et al., 2014). Next to macrophage attraction, FBGCs regulate the FBR through interaction with T cells (Chang et al., 2009). During FBRs, newly recruited T cells attach to macrophages and FBGCs than to the surface of the foreign particle. After docking, the recruited T cells become activated which may be mediated by FBGCs as they express several cytokines and inflammatory surface molecules, including MHC molecules. Depending on the nature of the biomaterial, FBGCs express a different repertoire or concentrations of inflammatory factors (Jones et al., 2007). Therefore, the FBR slightly differs between distinct biomaterials. For example, cells present on hydrophobic biomaterial produce small amounts of IL-1β and IL-6, whereas the production of these cytokines is enhanced on hydrophilic surfaces. The secretion of cytokines and chemokines is also time-dependent since FBGCs undergo a phenotypic switch (Hernandez-Pando et al., 2000; Jones et al., 2007). Initially, FBGCs merely produce pro-inflammatory cytokines, thereby stimulating inflammation. Later on, pro-inflammatory mediators are downregulated and the expression of anti-inflammatory cytokines increases (Jones et al., 2007). These phenotypic changes have been described in mice that were subcutaneously injected with nitrocellulose particles (Hernandez-Pando et al., 2000). Within the first 4 weeks, FBGCs produced high amounts of IL-1 and tumor necrosis factor (TNF)-α, two major cytokines involved in the recruitment and activation of immune cells. After 2 months, the inflammatory profile of FBGCs switched towards an anti-inflammatory phenotype, characterized by massive expression of TGF-β, a key factor in tissue repair and fibrosis (Hernandez-Pando et al., 2000; Pagán and Ramakrishnan, 2018; Weiskirchen et al., 2019). Apart from its capacity to promote fibroblast proliferation, TGF-β also stimulates fibroblasts, epithelial, and mesenchymal cells to produce extracellular matrix components, like collagen and fibronectin (Mornex et al., 1994; Wynn and Ramalingam, 2012; Pagán and Ramakrishnan, 2018; Weiskirchen et al., 2019). As aforementioned, FBGCs could be involved in the encapsulation of foreign bodies, which is characteristic for FBRs (Sheikh et al., 2015). However, Kyriakides et al. reported that the production of TGF-β is unaltered and that extensive fibrosis also takes place when FBGC formation is impaired, suggesting that other immune cells, such as macrophages, suffice to establish the fibrous capsule (Kyriakides et al., 2004).

## Langhans Giant Cells Phenotype, Functions, and Biological Activities

LGCs are circular or ovoid shaped and contain generally less than 20 nuclei, arranged in a circular or horseshoe pattern along the cell border (Anderson, 2000; Quinn and Schepetkin, 2009; Sakai et al., 2012; Pagán and Ramakrishnan, 2018; Wang et al., 2020). They were first described in 1868 by Theodor Langhans in his studies of granulomas in tuberculosis (Mizuno et al., 2001; Helming et al., 2009; Pagán and Ramakrishnan, 2018). Subsequently, LGCs have been described in various infectious and non-infectious granulomatous conditions (Sakai et al., 2012; Wang et al., 2020).

### Langhans Giant Cells in Infectious Diseases

In infectious diseases, LGCs are formed when individual macrophages fail to eradicate persistent pathogens (Pagán and Ramakrishnan, 2018). Because of their increased cell size compared to macrophages, it could be assumed that LGCs dispose better phagocytic properties (Rose et al., 2014). However, the phagocytic capacity of IL-15 induced LGCs for bacille Calmette-Guérin and *M. leprae* bacteria is similar to that of macrophages (Wang et al., 2020). Lay et al. even reported that human LGCs, derived from M. tuberculosis-induced granulomas, are unable to phagocytose beads coated with *M. tuberculosis* antigens (Lay et al., 2007). The impaired phagocytic capacity of LGCs was associated with downregulation of the mannose receptor and CD11b (a subunit of the C3 receptor), two phagocytic receptors. Taken together, these findings suggest that LGCs cannot to be considered as superior phagocytes.

The killing capacities of LGCs to pathogens remain a matter of debate as different research groups reported conflicting results. Yasui et al. demonstrated that LGCs produce decreased levels of superoxide anions, which may enable *M. tuberculosis* to survive within granulomas (Yasui et al., 2011). On the other hand, LGCs may have better fungicidal capacities than macrophages (do Nascimento et al., 2011; Enelow et al., 1992). Enelow et al. demonstrated that LGCs stimulated with phorbol myristate acetate, display enhanced fungicidal capacities against *C. albicans* due to elevated oxidative activity (Enelow et al., 1992). It is worth noting that Enelow et al. differentiated human monocytes into LGCs by stimulation with IFN-γ and IL-3, but that Yasui et al. did not use IFN-γ or IL-3 for the production of LGCs (Enelow et al., 1992; Yasui et al., 2011). As IFN-γ and IL-3 are able to promote superoxide anion production by monocytes (Jendrossek et al., 1993), the different microbicidal activity of LGCs in both reports might be due to differences in the differentiation protocol.

#### Role of LGCs in *Mycobacterium tuberculosis* Infection

**FIGURE 4:**
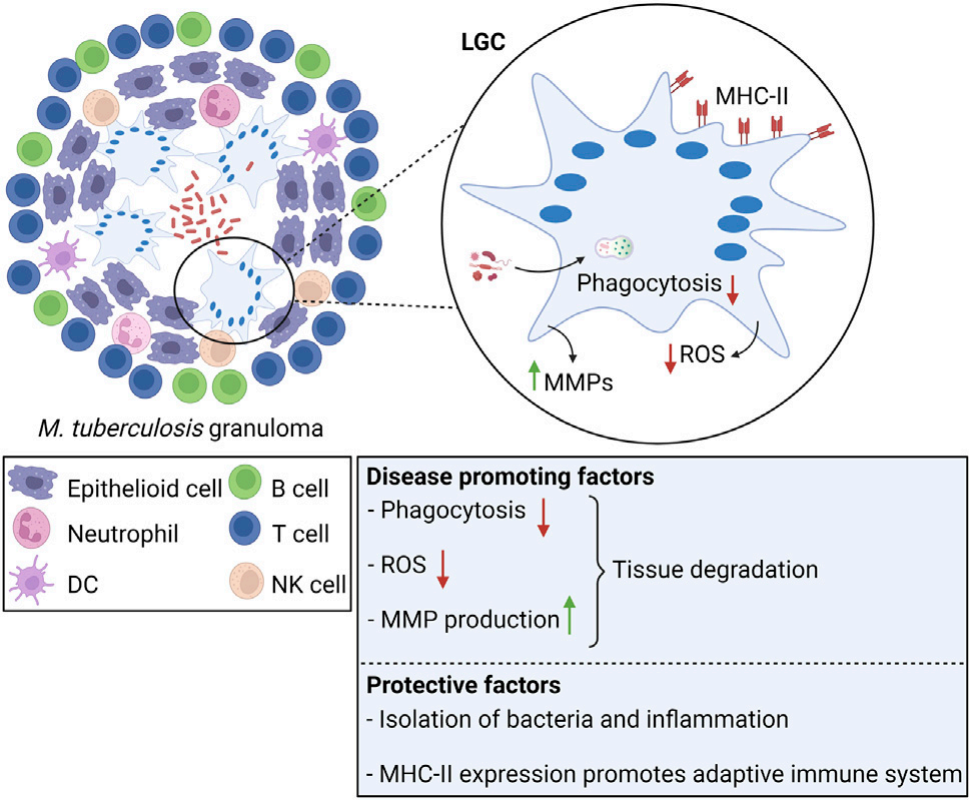
Langhans giant cells in tuberculosis. LGCs are a hallmark of M. tuberculosis-induced granulomas. In tuberculosis, LGCs exhibit both protective and disease promoting factors. Clearance of *M. tuberculosis* is inefficient since LGCs cannot mediate bacterial uptake or sufficient ROS production for pathogen killing. Additionally, LGCs secrete MMPs leading to tissue destruction. On the other hand, LGCs are involved in isolation of bacteria and inflammation from the surrounding tissue. LGCs highly express MHC-II molecules, suggesting that they promote the adaptive immune response through antigen presentation.

LGCs are most studied within the context of tuberculosis (Figure 4). Although LGCs are inefficient in destroying *M. tuberculosis*, either through phagocytosis or through production of microbicidal oxidants (Lay et al., 2007; Yasui et al., 2011), they still may exert protective functions. Within the granulomatous structure, LGCs form a barrier that shields *M. tuberculosis* from the rest of the body, and prevent cell-to-cell spread and growth of bacteria (Byrd, 1998; Brooks et al., 2019). Furthermore, LGCs express high amounts of MHC-II molecules, enabling them to function as antigen presenting cells, capable to prime the adaptive immune response (Lay et al., 2007). Conversely, tuberculosis-associated LGCs produce MMPs thereby inducing tissue destruction (Helming et al., 2009). LGCs may be involved in the inflammatory reaction of the granuloma through the production of cytokines and chemokines. In a murine model for pulmonary tuberculosis, immunostaining for TNF-α and IL-1α, two cytokines that have been reported to be crucial for granuloma formation (Huaux et al., 2015; Pereira et al., 2018), was strongly positive in LGCs (Hernandez-Pando et al., 1997). In addition to initiation and maintenance of granulomas, LGCs may also be involved in resolution of inflammation and development of fibrosis. As for FBGCs, LGCs undergo a phenotypic switch with time, characterized by reduced expression of TNF-α and IL-1β, and simultaneous upregulation of TGF-β, a major regulator of tissue repair and fibrosis (Hernandez-Pando et al., 1997; Wynn and Ramalingam, 2012).

Although LGCs are a major morphological characteristic of infectious granulomas, their specific biological activity and function still needs further elucidation.

### Langhans Giant Cells in Non-Infectious Diseases

The role of LGCs in non-infectious diseases is less understood. LGCs are a pathological hallmark in granulomatous inflammatory diseases, such as sarcoidosis and Blau syndrome (Okamoto et al., 2003; Janssen et al., 2012; Sakai et al., 2012; Wouters et al., 2014). Although these disorders have no infectious cause and are considered auto-inflammatory granulomatous conditions, DNA fragments of *Mycobacteria* are occasionally found within the granulomatous lesions through PCR-based detection methods (Dow and Ellingson, 2010; Lee et al., 2019; Poline et al., 2020). Whether the LGC-granulomas are formed in response to the bacteria or whether the lesions form the ideal environment for the pathogen to survive remains unclear.

In some cases, LGCs can be found in close proximity to cancer cells (Bigotti et al., 2002) and may be involved in the clearance of tumor cells. Wang et al. reported that LGCs are able to phagocytose malignant cells in esophageal cancer (Wang et al., 2021). The precise role of LGCs or granulomas within malignant diseases is not elucidated. In Hodgkin disease, the presence of granulomas is associated with more beneficial outcomes (Pagán and Ramakrishnan, 2018), whereas intravascular granulomas may worsen the clinical outcome in testicular seminomas (Downes et al., 2016).

## Formation of Multinucleated Giant Cells Through Macrophage Fusion

Throughout the decades, scientists have been searching for the mechanism of MGC formation and several hypotheses have been proposed, the most plausible being endoreplication, “frustrated” phagocytosis, and cell fusion (McNally et al., 1996; DeFife et al., 1997; McNally and Anderson, 2005; Herrtwich et al., 2016). Endoreplication refers to the failure of the actual cell division after the nucleus has been replicated (Herrtwich et al., 2016). “Frustrated” phagocytosis was suggested to be a mechanism of multinucleation after the observation that proteins involved in phagocytosis seemed to be important for MGC formation as well (McNally et al., 1996; DeFife et al., 1997; McNally and Anderson, 2005). In this model of macrophage fusion, MGCs originate from macrophages that phagocytose particles in close proximity to other macrophages (DeFife et al., 1999). When individual macrophages are unable to engulf a foreign particle, this may eventually lead to “frustrated” phagocytosis and internalization of surrounding macrophages (DeFife et al., 1999). Although it is difficult to exclude the above mechanisms, it is nowadays accepted that MGCs originate from fusion of cells of the monocyte-macrophage lineage.

**FIGURE 5:**
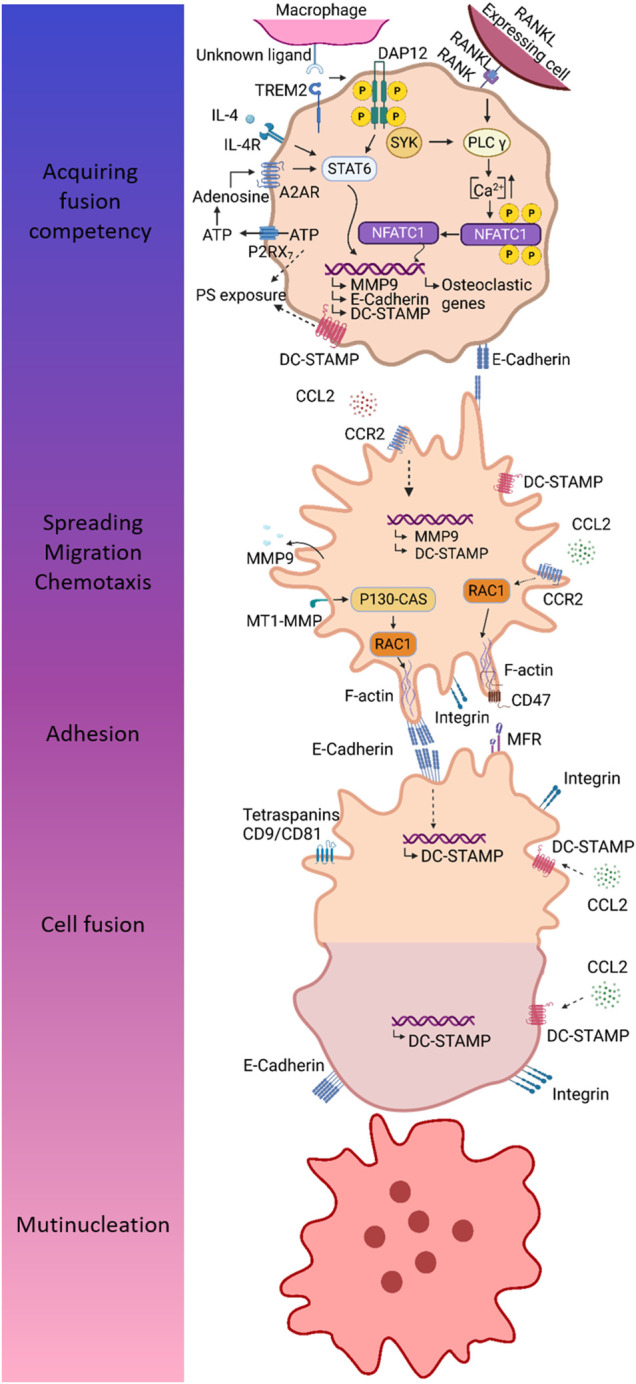
Different steps in the multinucleation process mainly based on findings in osteoclasts. MGCs originate from macrophage fusion, a multi-step process comprising acquirement of fusion competency, chemotaxis and adhesion, and finally cell fusion resulting into multinucleation. Fusion competency is accomplished through upregulation of fusogens downstream of signaling through so-called prefusion mediators. DAP12 and its associated receptor TREM2 are well-established prefusion mediators. After binding of its unknown ligand, TREM2 associates with DAP12 leading to phosphorylation of DAP12 and downstream signaling via SYK. In IL-4 induced fusion, DAP12-mediated signaling results in activation of STAT6 and subsequent induction of several fusogens, including MMP9, E-cadherin, and DC-STAMP. During osteoclastogenesis, combined action of RANK- and DAP12-mediated signaling leads to Ca^2+^ signaling downstream of PLCγ, resulting in an increase of intracellular Ca^2+^ concentration enabling translocation of NFATc1 to the nucleus. Fusogens are induced by combined action of P2RX7 and A2A receptor. ATP release through P2RX7 provides extracellular ATP for biosynthesis of adenosine, which in turn induces fusion competency by binding to A2A receptor. Fusing macrophages are characterized by exposure of PS, a lipid that is normally localized in the inner membrane leaflet. The mechanism of PS externalization may involve P2RX7 and DC-STAMP. Macrophage chemotaxis is driven by CCL2 and is essential to bring the membranes of two individual cells in close proximity for fusion. Next to chemotaxis, CCL2 is hypothesized to mediate the induction of fusogens, including MMP9 and DC-STAMP. In order to allow cell migration and subsequent cell adhesion, macrophages undergo cytoskeletal arrangements, which are mediated by RAC1, a major regulator of the cytoskeleton. It has been established that MT-MMP1 activates RAC1. Furthermore, CCL2 signaling has also been associated with RAC1 activation. Homotypic cell-cell adhesion is mediated by integrins and E-cadherin. The latter might also be involved in induction of fusogens, such as DC-STAMP. Cell-cell attachment is dependent on interaction between CD47 and MFR. Once macrophages are firmly attached to each other, the real fusion process can proceed. DC-STAMP is considered a main fusion regulator; however, its ligand and mechanism of action remain to be defined. Several putative ligands for DC-STAMP has been proposed, including CCL2, MFR, and CD47.

Macrophage fusion is a complex, not well understood multistep process. Before the real fusion process can be initiated, macrophages have to undergo a series of events: acquirement of a fusion competent state, chemotaxis and adhesion, and finally fusion (Brooks et al., 2019; Faust et al., 2019). Although all derived from macrophage fusion, distinct MGC subtypes are induced by different stimuli (Anderson, 2000). Combined action of M-CSF and RANKL has been well established for fusion of macrophages into osteoclasts (Asagiri and Takayanagi, 2007). IL-4 and IL-13 are efficient inducers of FBGCs *in vitro* (Anderson, 2000; Yang et al., 2014), whereas biomaterial implantation leads to generation of FBGCs *in vivo* (Yang et al., 2014). Fusion of macrophages into LGCs is triggered by cytokines, such as combinations of IFN-γ plus IL-3 or GM-CSF (Anderson, 2000), pathogens and derivates, including *M. tuberculosis* and muramyl dipeptide (Mizuno et al., 2001), and concanavalin A (Sakai et al., 2012). Whereas the stimuli vary between the different MGC subtypes, it is supposed that the actual fusion process relies on a common fusion machinery (Pereira et al., 2018). It should be noted that our insights in macrophage fusion are incomplete and highly dependent on MGC subtype, stressing the need for additional and in-depth analysis of this phenomenon in all MGCs. Especially our knowledge on LGC formation remains unsatisfactory, reflected by sparse literature on this topic. In the next section, we provide an overview of the current knowledge on the molecular pathways and key molecules involved in macrophage fusion (Figure 5).

### Fusion Competency by Upregulation of Fusogens

Initially, macrophages have to become fusion competent, through upregulation of essential fusogens (Pereira et al., 2018), which are proteins required for cell fusion (Brukman et al., 2019). Fusogens are induced by signaling through so-called prefusion mediators expressed on macrophages, including DNAX-activating protein of 12 kDa, triggering receptor expressed on myeloid cells 2 (TREM2), and P2X7 receptor (P2RX7) (Pereira et al., 2018).

#### DAP12 – TREM2 Interaction

DNAX-activating protein of 12 kDa (DAP12) is a transmembrane adaptor protein (Paloneva et al., 2003) predominantly expressed on NK cells (Takaki et al., 2006; Tessarz and Cerwenka, 2008) and on cells of the myeloid lineage (Takaki et al., 2006), including monocytes (Tessarz and Cerwenka, 2008), macrophages, neutrophils (Tessarz and Cerwenka, 2008), and DCs (Humphrey et al., 2004). DAP12 is expressed as a homodimer at the cell surface, where it associates with DAP12-associated receptors to exert its function (Humphrey et al., 2004). In DAP12-deficient mice, bone mass is increased (Humphrey et al., 2004), suggesting a role for DAP12 in osteoclast formation and/or function. Osteoclast fusion is inefficient in monocytes derived from patients with loss-of-function mutations in DAP12 (Paloneva et al., 2003) and in bone marrow macrophages derived from DAP12-deficient mice (Humphrey et al., 2004). By contrast, stimulation of DAP12 in RAW264.7 cells (a monocyte/macrophage cell line derived from mice) enhances fusion, resulting in more multinucleated osteoclasts with increased cell size and number of nuclei (Humphrey et al., 2004). A role for DAP12 in fusion is not exclusive to osteoclasts as Helming et al. demonstrated that IL-4 induced fusion is also impaired in human macrophages silenced for DAP12 (Helming et al., 2008). Triggering receptor expressed on myeloid cells 2 (TREM2) functions as a DAP12-associated receptor in osteoclasts (Takaki et al., 2006; Peng et al., 2010; Zou and Teitelbaum, 2015) and has also been reported to mediate osteoclast multinucleation (Paloneva et al., 2003). It is hypothesized that macrophages themselves express a TREM2 ligand and thus that TREM2/DAP12-mediated signaling relies on the interaction between macrophages (Peng et al., 2010). During osteoclastogenesis, TREM2 is upregulated and its expression positively correlates with the number of nuclei in mature osteoclasts (Humphrey et al., 2006). Stimulation of TREM2 enhances multinucleation (Humphrey et al., 2004), whereas silencing or blockade of TREM2 inhibits osteoclast multinucleation (Humphrey et al., 2006; Peng et al., 2010). Finally, human PBMCs derived from TREM2-deficient patients, show impaired fusion when differentiated into osteoclasts (Paloneva et al., 2003). Together, these results demonstrate that combined action of DAP12 and TREM2 is important to macrophage fusion. After recognition of its unknown ligand, TREM2 associates with DAP12 leading to the phosphorylation of the immunoreceptor tyrosine-based activation motif (ITAM) in the cytoplasmic domain of DAP12 (Paloneva et al., 2003; Pereira et al., 2018). Once phosphorylated, DAP12 interacts with the cytoplasmic protein tyrosine kinases SYK and ZAP70, thereby triggering several downstream pathways (Paloneva et al., 2003). Although both FBGCs and osteoclasts rely on DAP12/TREM2-signaling, distinct downstream pathways are involved to acquire a fusion competent state (Pereira et al., 2018). In FBGC precursors, cooperation of IL-4 and ITAM signaling results in the activation of transcription factor STAT6 which in turn induces the expression of essential fusogens, including E-cadherin, DC-STAMP, and MMP9 (Moreno et al., 2007; Helming and Gordon, 2009; Van den Bossche et al., 2012; Pereira et al., 2018). In osteoclast precursors, recruitment of SYK to DAP12 leads to Ca^2+^ signaling downstream of PLCγ (Koga et al., 2004). Ca^2+^ signaling enhances the induction of nuclear factor of activated T cells c1 (NFATc1), the master transcription factor of osteoclastogenesis, through an autoamplification mechanism (Koga et al., 2004; Kameda et al., 2013).

It is worth mentioning that DAP12-depleted mice still form multinucleated osteoclasts *in vivo*, whereas multinucleation is severely inhibited *in vitro* (Humphrey et al., 2004). Therefore, other mechanisms compensate for fusion in case of DAP12 deficiency *in vivo*. Zou et al. demonstrated that FcRγ, another ITAM-containing signaling adaptor that activates SYK signaling, can rescue osteoclast multinucleation in absence of DAP12 (Zou and Teitelbaum, 2015).

#### P2X7 Receptor

P2RX7 is an ATP-gated ion channel that belongs to the family of P2X purinergic receptors (Di Virgilio et al., 1999; Gartland et al., 2003; Lemaire et al., 2006). Although P2RX7 functions as a cation-selective channel under transient stimulation with ATP, the receptor transforms into a nonselective pore for hydrophilic molecules of a molecular mass up to 900 Da upon repetitive ATP stimulation (Di Virgilio et al., 1999; Falzoni et al., 2000). During macrophage fusion, P2RX7 localizes at sites of cell-cell contact in podosomes (Falzoni et al., 2000; Lemaire et al., 2012), indicating that the receptor plays a role in macrophage fusion. The involvement of P2RX7 in fusion is further supported by the finding that mouse J774 macrophages expressing high levels of P2RX7 spontaneously form MGCs (Chiozzi et al., 1997; Di Virgilio et al., 1999; Falzoni et al., 2000). Previously, it was assumed that P2RX7 drives fusion through the formation of a “fusion pore”, thereby connecting the cytoplasm of two neighboring cells (Falzoni et al., 2000; Lemaire et al., 2012). This hypothesis was supported by the finding that P2RX7 pore-forming activity is required for macrophage fusion (Lemaire et al., 2012). Blockade of the pore activity with P2RX7 neutralizing antibodies or with the receptor antagonist oxidized ATP, impairs fusion of human and murine macrophages (Chiozzi et al., 1997; Di Virgilio et al., 1999; Falzoni et al., 2000; Gartland et al., 2003; Steinberg and Hiken, 2007). Additionally, polymyxin B, a natural cationic peptide that potentiates pore activity in response to ATP, facilitates fusion of HEK293 cells transfected with P2RX7 (Lemaire et al., 2006). By contrast, HEK293 cells transfected with a truncated form of P2RX7 that lacks ATP-induced pore-forming activity, show impaired fusion even in presence of polymyxin B. Interestingly, hydrolyzation of extracellular ATP, by hexokinase or apyrase, accelerates MGC formation, which conflicts with the idea of ATP-induced pore formation during fusion (Di Virgilio et al., 1999; Pellegatti et al., 2011). These observations may be explained by receptor desensitization when stimulated with high ATP concentrations as degradation of ATP by hexokinase or apyrase might restore the responsiveness of P2RX7 (Falzoni et al., 2000). More recently, Pellegatti et al. proposed a new concept in which P2RX7 solely functions as a receptor for local ATP release to provide extracellular ATP for biosynthesis of adenosine, the actual fusogen (Pellegatti et al., 2011). Indeed, small amounts of extracellular ATP restore fusion in presence of anti-P2RX7 antibodies. Moreover, extracellular degradation of adenosine abolishes fusion, whereas addition of adenosine has the opposite effect. During macrophage fusion, adenosine likely acts at the adenosine receptor A2A as pharmacological inhibition of the A2A receptor reduces fusion, whereas A2A receptor agonists increase fusion. The finding that combined action of a P2RX7 inhibitor and A2A receptor agonists allows fusion, indicates that proper adenosine supply is required for MGC formation.

### Chemotaxis

Chemotaxis and migration of macrophages towards each other is a crucial step before cell-cell fusion. CCL2 and its receptor CCR2 mediate chemotaxis of monocytes/macrophages (Li et al., 2007; Khan et al., 2016; Guicciardi et al., 2018) and their role in macrophage fusion has been established. CCL2 potentiates RANKL-induced osteoclast formation in human and mice (Kim et al., 2005; Li et al., 2007) and even induces the formation of osteoblast-like cells in absence of RANKL or when NFATc1 is inhibited (Kim et al., 2005). Osteoclast-like cells are multinucleated, but lack bone resorbing activity, suggesting that CCL2 stimulates the fusion process rather than osteoclast differentiation (Kim et al., 2005). Furthermore, CCL2 or CCR2-deficient mice display impaired osteoclast fusion *in vivo* and *in vitro* (Miyamoto et al., 2009; Khan et al., 2016) and exogenous CCL2 restores fusion in cultures derived from CCL2-deficient mice (Khan et al., 2016). Several findings illustrate the involvement of CCL2 and CCR2 in FBGC formation as well. Blocking of CCL2, either by an inhibitory peptide or neutralizing antibodies, diminishes IL-4 induced fusion of human monocytes (Kyriakides et al., 2004). In addition, bone marrow cultures derived from CCL2 or CCR2-deficient mice, display impaired FBGC multinucleation, whereas exogenous CCL2 rescues fusion in CCL2-deficient cultures (Khan et al., 2016). *In vivo* macrophage fusion on implanted biomaterials is also abrogated in CCL2-deficient mice (Kyriakides et al., 2004; Skokos et al., 2011), probably due to reduced macrophage accumulation and migration (Skokos et al., 2011). Next to chemotaxis, CCL2 is thought to contribute to the fusion competency through induction of essential fusogens, including DC-STAMP and MMP9 (Helming and Gordon, 2009; Miyamoto et al., 2009; Skokos et al., 2011), and through activation of Rac1 (Skokos et al., 2011), a major cytoskeleton regulator (Touaitahuata et al., 2014).

### Adhesion

After chemotaxis, macrophages attach to each other to bring their membranes in close proximity prior to cell fusion. The homotypic cell-cell contacts are partly mediated by E-cadherins and some studies elucidate that integrins might be involved as well (Helming and Gordon, 2009).

#### E-Cadherin

E-cadherin belongs to the family of the cadherins, which are transmembrane or membrane-associated glycoproteins that mediate calcium-dependent cell-cell adhesions (Mbalaviele et al., 1995; van Roy and Berx, 2008). During FBGC formation, E-cadherin is upregulated at the macrophage cell surface downstream of IL-4 or IL-13 signaling (Moreno et al., 2007; Van den Bossche et al., 2009, 2012; Wanat et al., 2014; Fiorino and Harrison, 2016), suggesting for a role in macrophage fusion. Moreover, E-cadherin neutralizing antibodies impair IL-4 induced macrophage fusion, especially in early stages of differentiation (Moreno et al., 2007), and fusion is diminished in macrophages derived from E-cadherin deficient mice (Van de Bossche et al., 2009; Van de Bossche et al., 2012). Fusion-efficient macrophages are characterized by membrane extension, called podosomes, which form zipper-like structures at cell fusion sites (Balabiyev et al., 2020). E-cadherin is probably implicated in the generation of these zipper-like structures as neutralizing antibodies disrupt formation of these structures and subsequent fusion (Balabiyev et al., 2020). E-cadherin is also upregulated during osteoclastogenesis, especially at early differentiation stages, suggesting that E-cadherin is involved in early osteoclastogenesis (Fiorino and Harrison, 2016). Blocking of E-cadherin, either by neutralizing antibodies (Mbalaviele et al., 1995; Fiorino and Harrison, 2016; Sun et al., 2020) or synthetic peptides containing the cell adhesion recognition sequence of cadherins (Mbalaviele et al., 1995), diminishes osteoclast fusion (Mbalaviele et al., 1995; Fiorino and Harrison, 2016), whereas E-cadherin overexpression transiently accelerates fusion (Fiorino and Harrison, 2016). Although E-cadherin is upregulated at membrane extensions and cell-cell contact sites, the protein is not detected at fusion sites, indicating that it does not make part of the fusion machinery (Fiorino and Harrison, 2016). Instead, E-cadherin neutralizing antibodies impact migration and expression of fusogens, including DC-STAMP, suggesting that cell-cell contact mediated through E-cadherin alters macrophage proliferation and induce the transition to cell migration and fusion.

#### Integrins

Integrins are a superfamily of heterodimeric transmembrane proteins comprised of α and β subunits, which mediate both cell-extracellular matrix and cell-cell adhesions (Campbell and Humphries, 2011; Aghbali et al., 2017). The integrin subunits β1, β2, and β5 have been reported to contribute to FBGC formation. Anti-β1 and anti-β2 antibodies impair the adhesion of human monocyte/macrophage to biomaterials, an essential prerequisite for FBGC formation (McNally and Anderson, 2002). Although blockage of β1 dramatically reduces the number of adherent monocyte/macrophages, some FBGCs are still formed, indicating that fusion competency is remained. By contrast, β2 neutralizing antibodies also abolishes fusion, suggesting that β2 mediates both intercellular and cell-biomaterial interactions during FBGC formation. αMβ2 is involved in IL-4 induced fusion of murine peritoneal macrophages both *in vivo* and *in vitro*. Indeed, blocking of αMβ2 results in less FBGCs formation and the remaining FBGCs are smaller in size and contain less nuclei (Podolnikova et al., 2016). αDβ2 might also contribute to IL-4 induced fusion of murine macrophages, however to a lower extend as αMβ2. Finally, IL-4 has been reported to increase the expression of β5 on human monocytes, suggesting a role for this integrin in FBGC fusion (Aghbali et al., 2017). Integrins are also involved in the formation of multinucleated osteoclasts (Rao et al., 2006). It has been found that α9β1 contributes to osteoclast multinucleation, reflected by reduced osteoclast formation in human bone marrow cultures treated with anti-α9 antibodies and smaller osteoclast size in α9-deficient mice.

#### Macrophage Fusion Receptor and CD47

Macrophage fusion receptor (MFR), also called signal regulatory protein α (SIRPα), and CD47 are transmembrane proteins that belong to the superfamily of immunoglobulins (Igs) (Han et al., 2000; Gautam and Acharya, 2014; Zhang et al., 2020). It was demonstrated that MFR and CD47 are induced at the onset of fusion both *in vivo* and *in vitro*, suggesting that these proteins contribute to macrophage fusion (Saginario et al., 1995; Saginario et al., 1998; Han et al., 2000; Podolnikova et al., 2019). Moreover, blocking of MFR or CD47, either by monoclonal antibodies or engineered proteins containing the extracellular domain of MFR or CD47, impairs fusion. Finally, IL-4 induced fusion is abrogated in RAW264.7 cells depleted for MRF by short hairpin (sh)RNA (Podolnikova et al., 2019). Han et al. proposed a model for MFR and CD47 mediated fusion. In order to guarantee cell-cell attachment, CD47 may initially interact with a “long” form of MFR on the neighboring macrophage, consisting of an extracellular immunoglobulin V domain and two adjacent immunoglobulin constant domains (Han et al., 2000; Vignery, 2005a). Afterwards, CD47 switches to a poorly expressed “short” form of MFR, only containing the immunoglobulin V domain. This homotypic interaction brings the plasma membranes of the two cells to a distance of 5–10 nm, allowing cell fusion (Han et al., 2000; Vignery, 2000, 2005a). MFR can also interact with the integrin CD11b contributing to fusion (Podolnikova et al., 2019). In macrophages, MFR and CD11b are detected at sites of cell-cell contact. Furthermore, IL-4 induced fusion has been observed in co-cultures of MFR and CD11b expressing HEK293 cells. Despite the findings favoring a role for MFR and CD47 in macrophage fusion, van Beek et al. reported that the number of nuclei remains unaltered in mice lacking the cytoplasmic signaling region of MFR, indicating that MFR or at least MFR signaling is dispensable for osteoclast fusion (van Beek et al., 2009). Additionally, osteoclasts derived from bone marrow cells of CD47 KO mice or differentiated in presence of MFR neutralizing antibodies do not differ in size or number of nuclei (Lundberg et al., 2007). Together, these findings suggest that MFR and CD47 may be involved in macrophage fusion other that osteoclast formation.

### Cytoskeletal Alterations

Cytoskeletal alterations are implicated in many aspects of macrophage fusion, including chemotaxis/migration, adhesion, the actual fusion process, and cellular reorganization (Wang et al., 2008, 2015; Pereira et al., 2018). Fusing macrophages are characterized by actin-based membrane protrusions and these structures have been shown to induce fusion (Wang et al., 2015; Faust et al., 2019). Indeed, almost all fusion events take place at membrane protrusions and impaired formation of these structures prevents macrophage fusion (Faust et al., 2019). The actin cytoskeleton is a dynamic structure that is constitutively remodeled by actin organizing proteins (Fritzsche et al., 2017). An important group of actin organizers are the Rho-related small GTPases with Rac1, Cdc42, and RhoA as the best characterized members to mediate macrophage fusion (Wang et al., 2008; Touaitahuata et al., 2014). Pharmacological inhibition or genetic depletion of Rac1 impairs the fusion of mouse macrophages into FBGCs and osteoclasts (Jay et al., 2007, 2010; Wang et al., 2008; Takito et al., 2015). Furthermore, release of Rac1 inhibitor from implanted biomaterials reduces FBGC formation *in vivo* (Jay et al., 2007). Next to Rac1, Cdc42 positively regulates fusion of mouse macrophages into FBGCs and osteoclasts (Leung et al., 2010; Faust et al., 2019; Park et al., 2019). The effect of RhoA on macrophage fusion is less straightforward. RhoA activation leads to more but smaller osteoclasts, whereas inhibition of its downstream effector Rho kinase elevates the formation of large osteoclasts (Takito et al., 2015). By contrast, inhibition of Rho kinase induces more but smaller FBGCs (Jay et al., 2007). Contradictory results have also been published on the involvement of actin in macrophage adhesion and migration prior to fusion. DeFife et al. reported that cytochalasin B and D, two substances that disrupt actin dynamics, prevent the formation of multinucleated FBGCs without affecting macrophage adhesion, spreading, and motility (DeFife et al., 1999). Inhibition of Rac1 reduces macrophage fusion on implanted biomaterials *in vivo* without affecting macrophage recruitment (Jay et al., 2007). The formation of FBGCs in response to implanted biomaterials is also affected in Cdc42-deficient mice, but depletion of Cdc42 has no effect on macrophage recruitment or adhesion (Faust et al., 2019). By contrast, osteoclast precursors from Rac1-depleted mice display, next to impaired fusion, reduced cell spreading and motility (Wang et al., 2008). Furthermore, cytochalasin D abolishes macrophage migration during osteoclastogenesis (Wang et al., 2015). Together, these illustrations suggest that macrophage adhesion, spreading and motility can be maintained during FBGC formation even though the actin network is dysregulated, whereas this is not the case during osteoclastogenesis.

### Lipid Alterations

During macrophage fusion, the lipid composition of the cytoplasmic and extracellular plasma membrane leaflet is altered to allow fusion. Phosphatidylserine (PS) is normally located in the cytoplasmic membrane leaflet (Shin and Takatsu, 2020), but is externalized during fusion (Verma et al., 2018; Kang et al., 2020). The importance of PS exposure during osteoclast multinucleation is illustrated by PS neutralization at the cell surface, either by antibodies or PS-binding protein, leading to diminished fusion (Verma et al., 2018; Kang et al., 2020; Whitlock and Chernomordik, 2021). Moreover, silencing or pharmacological inhibition of lipid transporters involved in outer translocation of PS, prevents PS externalization and subsequent fusion of osteoclast precursors. DC-STAMP and P2RX7, two well-described fusion proteins, might be implicated in PS exposure during macrophage fusion as PS externalization is abrogated by anti-DC-STAMP antibodies and brief activation of P2RX7 channels causes reversible PS exposure on the cell surface (Helming and Gordon, 2009; Lemaire et al., 2012; Verma et al., 2018). During FBGC formation, fusion probably depends on recognition of PS by the lipid receptor CD36 (Helming et al., 2009). CD36 localizes at sites of cell contact and IL-4 induced fusion of macrophages derived from CD36-deficient mice is impaired. Although CD36 is required for FBGC formation, the receptor is dispensable for osteoclast fusion as anti-CD36 antibodies nor CD36 KO macrophages display reduced osteoclast multinucleation.

Next to PS, phosphatidylethanolamine (PE) is also involved in osteoclast formation (Irie et al., 2017; Whitlock and Chernomordik, 2021). Under normal conditions, PE is located in the inner plasma membrane leaflet (Shin and Takatsu, 2020), but clusters in the extracellular leaflet at fusion sites during osteoclastogenesis (Irie et al., 2017). Moreover, inhibition of PE synthesis or translocation to the outer plasma membrane impairs PE exposure and osteoclast fusion (Irie et al., 2017).

### Fusion

After successful acquirement of fusion competency, chemotaxis and adhesion, and essential cytoskeletal and lipid alterations, the fusion process goes into its final execution. In this final stage, it is worth to mention a few key players, including dendritic cell-specific transmembrane protein (DC-STAMP), tetraspanins, and matrix metalloproteases (MMPs).

#### Dendritic Cell-Specific Transmembrane Protein

Dendritic cell-specific transmembrane protein (DC-STAMP) is a seven-transmembrane protein (Chiu et al., 2012) that is essential for macrophage fusion in human and mice (Yagi et al., 2005, 2006; Kim et al., 2008; Miyamoto, 2011; Zeng et al., 2013; Møller et al., 2020). The expression of DC-STAMP in human osteoclast precursors is positively correlated with the number of nuclei in mature osteoclasts (Møller et al., 2020). Moreover, suppression of DC-STAMP in human monocytes through lentivirus-mediated RNA interference impairs the formation of multinucleated osteoclasts (Zeng et al., 2013). Depletion of DC-STAMP in mice abrogates fusion of preosteoclasts into multinucleated osteoclasts both *in vivo* and *in vitro* (Yagi et al., 2005, 2006; Kim et al., 2008; Miyamoto, 2011) and reintroduction of DC-STAMP in osteoclast precursors derived from DC-STAMP-deficient mice rescues multinucleation (Yagi et al., 2005, 2006). Although osteoclast precursors isolated from DC-STAMP-deficient mice are unable to fuse, the expression of osteoclast markers, including TRAP (Yagi et al., 2005, 2006) and NFATc1, is not impaired (Yagi et al., 2006). Furthermore, ruffled borders and actin rings, typical morphological features of osteoclasts, are observed and cells exhibit minor bone-resorbing capacities (Yagi et al., 2005, 2006). These findings suggest that DC-STAMP is required for macrophage fusion without mediating osteoclast differentiation. Next to osteoclast multinucleation, DC-STAMP is indispensable for the formation of multinucleated FBGCs and LGCs (Yagi et al., 2005, 2006; Kim et al., 2008; Miyamoto, 2011; Sakai et al., 2012). Generation of multinucleated FBGCs is abolished in mice deficient for DC-STAMP *in vivo* and *in vitro* (Yagi et al., 2005, 2006; Kim et al., 2008; Miyamoto, 2011). Additionally, DC-STAMP is upregulated during LGC formation and siRNA-mediated knock-down of DC-STAMP inhibits fusion of human monocytes into LGCs (Sakai et al., 2012). Taken together, DC-STAMP is considered a common fusion mediator, yet different signaling mechanisms are responsible for induction of DC-STAMP in osteoclasts, FBGCs, and LGCs. During osteoclastogenesis, DC-STAMP is induced downstream of c-FOS and NFATc1 signaling (Yagi et al., 2007; Kim et al., 2008; Miyamoto, 2011; Sakai et al., 2012; Zhang et al., 2014), whereas PU.1 and NF-κB mediate the expression of DC-STAMP during FBGC formation (Yagi et al., 2007; Sakai et al., 2012), and probably NF-κB and MAP kinases during LGC formation (Sakai et al., 2012). It is speculated that DC-STAMP promotes macrophage fusion in a receptor-ligand fashion (Yagi et al., 2005), but the ligand for DC-STAMP required for macrophage fusion remains currently unknown (Quinn and Schepetkin, 2009). Since DC-STAMP shows structural similarities with chemokine receptors, it is speculated that a certain chemokine, such as CCL2, serves as ligand for DC-STAMP (Yagi et al., 2007; Quinn and Schepetkin, 2009). Other possible candidates include MFR and CD47. The exact mechanism by which DC-STAMP mediates macrophage fusion remains speculative as well. It is hypothesized that DC-STAMP induces the expression of fusogens (Zhang et al.), including MFR, and therefore indirectly mediates macrophage fusion (Vignery, 2005b).

#### Tetraspanins

Tetraspanins are four-span transmembrane proteins (Sangsri et al., 2020) that bind to one another and to a variety of other transmembrane proteins (Parthasarathy et al., 2009). Because of their ability to interact with several molecules, tetraspanins act as membrane organizers, mediating the generation of functional protein clusters in tetraspanin-enriched microdomains (Parthasarathy et al., 2009) of which some may modulate macrophage fusion. CD9 and CD81 are the best studied tetraspanins in the context of macrophage fusion, but whether they regulate fusion in a positive or negative manner remains controversial. For example, CD81 neutralizing antibodies support fusion of mouse macrophages infected with *B. thailandensis*, whereas inhibition of CD81 diminishes fusion upon infection with *B. pseudomallei* (Elgawidi et al., 2020; Sangsri et al., 2020). Additionally, CD9 neutralizing antibodies promote the formation of multinucleated osteoclast from murine bone marrow cells (Takeda et al., 2003), whereas blocking of CD9, either by neutralizing antibodies or small interfering RNA (siRNA), impairs the formation of multinucleated osteoclasts in RAW264.7 cells (Ishii et al., 2006). Although the effect of CD9 and CD81 on macrophage fusion remains unclear, CD63 seems to be a positive regulator (Takeda et al., 2003; Parthasarathy et al., 2009). CD63 is upregulated in human monocytes stimulated with Concanavalin A, and monoclonal antibodies directed against CD63 abrogate MGC formation. Tspan-5 and NET-6 have also been suggested to influence macrophage fusion as Tspan-5 is upregulated during osteoclastogenesis and NET-6 is downregulated (Iwai et al., 2007). Furthermore, silencing of Tspan-5 impairs fusion of RAW264.7 into multinucleated osteoclasts, whereas the opposite is true for NET-6.

#### Matrix Metalloproteases

Matrix metalloproteases (MMPs) compromise a family of endopeptidases known to degrade various extracellular matrix components (EMCs) (Gonzalo et al., 2010; Kim and Lee, 2020). MMPs may modulate macrophage fusion by degrading EMCs and/or cell-surface bound molecules involved in cell fusion (Jones et al., 2008). *In vitro* and *in vivo* fusion of macrophages into osteoclasts and FBGCs is abolished in mice deficient for MT1-MMP, a membrane-anchored MMP (Gonzalo and Arroyo, 2010; Gonzalo et al., 2010). Depletion of MT1-MMP affects macrophage spreading, formation of lamellipodia, and Rac1 activity, indicating that impaired migration lies at the basis of reduced fusion events in these cells (Gonzalo and Arroyo, 2010). MT1-MMP probably promotes fusion through its interaction with adaptor protein p130Crk-associated substrate (CAS), which in turn triggers Rac1 activation (Gonzalo and Arroyo, 2010; Gonzalo et al., 2010). Although MT1-MPP positively regulates macrophage fusion (Gonzalo and Arroyo, 2010; Gonzalo et al., 2010), MMP8 and MMP13 have been described to negatively impact fusion (Kim and Lee, 2020). RANKL decreases the expression of MMP8 and MMP13 in murine bone marrow macrophages, indicating that their down-regulation is required for proper osteoclastogenesis. Targeting these MMPs, either through siRNA or pharmacological inhibition, facilitates osteoclast fusion without altering differentiation (Kim and Lee, 2020). These findings are in contrast to data from Fu et al. who demonstrated that MMP13 promotes osteoclast formation starting from mouse bone marrow macrophages and that fusion is abrogated in macrophages derived from MMP13-deficient mice (Fu et al., 2016). Several reports indicate that MMPs also regulate FBGC formation. Pharmacological inhibition experiments suggested that MMP1, -8, -13, and -18 act as positive regulators of IL-4 induced fusion in human monocytes, whereas MMP2, -3, -9, and -12 do not influence fusion (Jones et al., 2008). Notwithstanding the observation that MMP9 is not implicated in human macrophage fusion, MMP9 neutralizing antibodies reduce IL-4 induced fusion of mouse macrophages and MMP9-deficient mice display reduced FBGC formation after implantation of biomaterials (Maclauchlan et al., 2009). The impaired fusion observed in MMP9-deficient mouse macrophages may result from defective cytoskeletal dynamics prior to cell fusion. The discrepancy on the effect of MMP-9 on macrophage fusion between human and mice may be explained by suboptimal specificity of pharmacological MMP inhibitors used in human experiments.

## Conclusion and Remaining Key Questions

MGCs are polykaryons that originate from fusion of cells of the monocyte-macrophage lineage and are subdivided into three main subtypes: osteoclasts, FBGCs, and LGCs (Anderson, 2000). Osteoclasts are well-known for their bone-resorbing capacities and increasing evidence is found that they contribute to multiple processes beyond bone resorption, including osteoblast stimulation, vasculogenesis and immune regulation (Novack and Teitelbaum, 2008; Le Goff et al., 2013; Cappariello et al., 2014; Drissi and Sanjay, 2016). FBGCs are formed during inflammatory reactions against foreign body material to remove particles that single macrophages cannot (Han et al., 2000; Vignery, 2000). Among all MGC subtypes, LGCs are the least characterized in terms of function and mechanism of formation. In infectious granulomatous diseases, LGCs are thought to be formed in response to a persistent pathogen as a final attempt to remove or isolate the irritant from the surrounding tissue (Byrd, 1998; Pagán and Ramakrishnan, 2018). In non-infectious granulomatous diseases, little is known about LGCs and multiple questions remain unresolved, like: What drives LGC formation and which mediators are essential to the fusion process? What are the mechanisms of macrophage fusion? Why do LGCs arise and what is their role in disease development and outcome? All MGCs have a common feature, namely a high number of nuclei. In osteoclasts, bone resorption is positively correlated with the number of nuclei, indicating that increased cell size empowers osteoclasts to degrade bone tissue (Yagi et al., 2006). In FBGCs, the acquisition of multiple nuclei may facilitate the engulfment of large particles (Milde et al., 2015). For LGCs, the functional consequences of multinucleation remain elusive. Furthermore, the nuclei of the three multinucleated subtypes are arranged in a unique fashion within the cytoplasm but to what extend the nuclear pattern influences the functionality of these cells has yet to be determined. Also, there is limited understanding of how these cells operate considering the many nuclei. In addition, it would be interesting to investigate whether MGCs are terminally differentiated or whether these cells still show plasticity. Implantation experiments in rats revealed that LGCs are the initial MGC subtype to be formed on the implanted particle, which over time fuse together to form FBGCs (van der Rhee et al., 1979; Smetana, 1987). It should be mentioned that these experiments date back from more than 30 years ago and that, to our knowledge, no recent reports about MGC plasticity have been published. In conclusion, despite being described over 150 years ago (Pagán and Ramakrishnan, 2018), multiple questions on MGCs remain unanswered, especially for LGCs. Since LGCs occur in a myriad of infectious and non-infectious diseases (Wang et al., 2020), in-depth analysis of the function, biological activity, and multinucleation of LGCs will provide us insights into the contribution of these cells to the pathogenesis of these diseases.
